# In Situ, Nitrogen-Doped Porous Carbon Derived from Mixed Biomass as Ultra-High-Performance Supercapacitor

**DOI:** 10.3390/nano14161368

**Published:** 2024-08-21

**Authors:** Yuqiao Bai, Qizhao Wang, Jieni Wang, Shuqin Zhang, Chenlin Wei, Leichang Cao, Shicheng Zhang

**Affiliations:** 1 Miami College, Henan University, Kaifeng 475004, China; byoungq@henu.edu.cn (Y.B.); 2038030073@henu.edu.cn (Q.W.); jieniwang@henu.edu.cn (J.W.); zhangshuqin@henu.edu.cn (S.Z.); wcl2000@henu.edu.cn (C.W.); 2 College of Chemistry and Molecular Sciences, Henan University, Kaifeng 475004, China; 3 Shanghai Key Laboratory of Atmospheric Particle Pollution and Prevention (LAP3), Department of Environmental Science and Engineering, Fudan University, Shanghai 200433, China; zhangsc@fudan.edu.cn

**Keywords:** hybrid biomass, porous carbon, heteroatom doping, supercapacitor

## Abstract

How to address the destruction of the porous structure caused by elemental doping in biochar derived from biomass is still challenging. In this work, the in-situ nitrogen-doped porous carbons (ABPCs) were synthesized for supercapacitor electrode applications through pre-carbonization and activation processes using nitrogen-rich pigskin and broccoli. Detailed characterization of ABPCs revealed that the best simple ABPC-4 exhibited a super high specific surface area (3030.2–3147.0 m^2^ g^−1^) and plentiful nitrogen (1.35–2.38 wt%) and oxygen content (10.08–15.35 wt%), which provided more active sites and improved the conductivity and electrochemical activity of the material. Remarkably, ABPC-4 showed an outstanding specific capacitance of 473.03 F g^−1^ at 1 A g^−1^. After 10,000 cycles, its capacitance retention decreased by only 4.92% at a current density of 10 A g^−1^ in 6 M KOH. The assembled symmetric supercapacitor ABPC-4//ABPC-4 achieved a power density of 161.85 W kg^−1^ at the maximum energy density of 17.51 Wh kg^−1^ and maintained an energy density of 6.71 Wh kg^−1^ when the power density increased to 3221.13 W kg^−1^. This study provides a mixed doping approach to achieve multi-element doping, offering a promising way to apply supercapacitors using mixed biomass.

## 1. Introduction

Energy, a concept that is often mentioned, has an irreplaceable place in our daily lives. Natural energy sources such as solar and wind energy could not be directly exploited. These sorts of energy forms were mostly turned into electric energy or other types of energy through the transformation of natural energy forms to suit the development needs of humans [1,2,3]. Until recent centuries, humans had mostly obtained energy from fossil fuels. However, the consumption of a huge number of these fuels could not accomplish long-term sustainable development, and environmental degradation was also severe [3,4,5,6]. To effectively solve the problems of energy structure and environmental pollution, one of the main research directions to efficiently reduce environmental pressure is the creation of energy storage materials utilizing biomass resources [7,8]. Among these, supercapacitors were widely utilized in railways, electronic communication, vehicles, and other industries as a type of energy storage material with high power density, high charging rate, extended cycle life, and environmental friendliness [9,10,11].

Electric double-layer capacitors (EDLCs), dummy capacitors, and hybrid supercapacitors are the three types of supercapacitors [12]. As an example, the charging and discharging process of the most popular electric double-layer capacitors (EDLCs) among these three types was realized by the storage and release of charges between inert carbon-based capacitors and electrolytes in the environment [12,13]. Scientists have worked for decades to develop conductive polymers, carbon-based compounds, transition metals, and other electrode materials [14,15]. Carbon-based capacitors, for example, could be made from a variety of biomass found in nature, including waste biomass from industrial and agricultural output [16,17]. This was not only because biomass includes a high concentration of carbon elements, but it could also significantly reduce consumption caused by additional environmental development and enable the recycling of certain biomass [11,18,19,20]. Furthermore, because of their high specific surface area (SSA), good pore size distribution, and low acquisition cost, many carbon-based materials prepared from biomass are frequently the first choice for making high-performance supercapacitors [21,22].

Numerous study findings exist on the synthesis of electrode materials from biomass carbon at the moment. With the continued investigation of these findings, it became clear that the essence that influenced the power of supercapacitors was ion transmission time [23]. Myriad approaches exist to improve this, such as creating carbon nanomaterials with improved pore structure using one-dimensional or two-dimensional plant materials [24] or realizing additional pseudo-capacitance effects by doping heteroatoms [25,26,27]. However, even if biomass materials with a better structure were chosen, the method of doping heteroatoms at the moment was primarily realized by the reaction of inorganic reagents [28], such as nitrogen atom doping by soaking in ammonia water solution or phosphorus and sulfur co-doping by soaking in phosphoric acid and sulfuric acid solution [29]. Depending on the kind, concentration, and reaction time of inorganic chemicals, these approaches more or less degraded the initial excellent carbon pore structure [30]. As a result, more research was required to achieve the preparation process of heteroatom doping while not degrading the carbon nanostructure of biomass.

Pigskin, a frequent material waste from the kitchen, was high in collagen and nitrogen. During pyrolysis, these proteins decompose to form nitrogen-doped carbon materials, which are known to improve electrical conductivity and enhance the electrochemical properties of the composite. Broccoli was a member of the Cruciferae family, which included plants with a high sulfur content [31]. The carbonization of broccoli contributes to the formation of a porous carbon structure. The presence of nitrogen from pigskin can prevent the collapse of these porous structures during pyrolysis, resulting in a more stable and interconnected pore network. This enhanced electrochemical properties, conductivity, and stability. These improvements make the composite material a promising candidate for various applications in energy storage and catalysis [32].

Herein, in situ, nitrogen-doped porous carbons (ABPCs) were prepared using nitrogen-rich pigskin and broccoli as precursors through pre-carbonization and activation processes for electrochemical application. The effect of different mixing ratios of pigskin and broccoli on the microstructure, elemental content, and electrochemical properties of the nanoporous biochar was investigated. The best sample, ABPC-4, possessed a super high specific surface area of 3030.2 m^2^ g^−1^ and abundant nitrogen (2.19 wt%) and oxygen content (11.75 wt%), showing an outstanding specific capacitance (473.03 F g^−1^ at a current density of 1.0 A g^−1^), excellent cycle stability (95.08% after 10,000 cycles at 10 A g^−1^ in 6 M KOH), and high energy storage performance (the maximum energy density was 17.51 Wh kg^−1^), which outperformed most electrode materials prepared from sole biomass. In this work, mixed biomass was converted into in situ nitrogen-doped porous carbons for high-performance supercapacitors, providing a facile way to treat food wastes and utilize biomass resources.

## 2. Materials and Methods

### 2.1. Materials and Chemicals

Broccoli was purchased from Yonghui Supermarket in Kaifeng City, and pork skin was purchased from the Kaifeng Tucheng Market (Kaifeng, Henan, China). The chemical reagents used in this study included potassium hydroxide (KOH, 85%) purchased from Tianjin Kermel Chemical Reagent Co., Ltd. (Tianjin, China) and hydrochloric acid (HCl, 38%) purchased from Sinopharm Chemical Reagent Co., Ltd. (Shanghai, China).

### 2.2. Preparation of Porous Carbon Materials from N-Rich Food Wastes

The broccoli was cut into pieces about 3 cm in length, washed with DI water to remove impurities on the surface of the broccoli, and dried in a blower oven at 60 °C for 12 h to obtain dehydrated broccoli. Put the processed dehydrated broccoli into a multifunctional pulverizer, grind it for 3 min, and then sieve the powder obtained after pulverization (~0.25 mm). The pig skin was scraped off the fat tissue with a knife, cut into 1 × 1 cm pieces, and degreased in 1 mol L^−1^ NaHCO_3_ solution for 12 h. The skin was washed with DI water and dried at 80 °C for 24 h. The dried skin was crushed in a multifunctional grinder for 3 min.

6 g of broccoli was mixed with different masses of pork skin (6, 3, 1.5, 0 g), transferred to a corundum boat, and heated up to 500 °C in a tube furnace under a nitrogen atmosphere at a rate of 3 °C min^−1^ for 1 h. After cooling to room temperature, the product was washed three times with anhydrous ethanol and three times with DI water, and then the solid product was dried overnight in a vacuum oven at 80 °C. The pre-carbonization biochar was recorded as BC, BPC-1, BPC-2 and BPC-4. The above biochar was weighed with KOH at a mass ratio of 1:4 and ground in an onyx mortar. The fully ground mixture was placed in a nickel crucible and then in a corundum boat, and then pyrolytically activated under a nitrogen atmosphere in a tube furnace (nitrogen flow rate of 25 mL min^−1^, pressure of 1 bar) at a heating rate of 3 °C min^−1^ to 700 °C and kept for 1 h. The mixture was cooled to room temperature. The temperature was increased to 700 °C at 3 °C min^−1^ and kept for 1 h. The samples were cooled to room temperature. The obtained samples were washed three times with 10 wt% HCl, and finally, they were washed with deionized water to neutrality and then filtered. The solid products were placed in a vacuum drying oven at 80 °C for overnight drying. The obtained multi-source nitrogen-enriched porous charcoal from kitchen wastes was named with ABC, ABPC-1, ABPC-2, and ABPC-4, with ABC indicating that the raw material was only broccoli, and 1, 2, and 4 indicating the mass ratios of broccoli and dried pork skin.

### 2.3. Material Characterization

Scanning electron micrographs of sample morphology were obtained by field emission scanning electron microscopy (SEM, Carl Zeiss, Oberkochen, Germany). The elemental composition of the samples was analyzed using a Vario EL cube (Elementar, Frankfurt, Germany). The X-ray photoelectron spectra (XPS) of the samples were obtained using AXIS ULTRA (Kratos, Manchester, UK). X-ray diffractograms from 5° to 80° were obtained from an X-ray powder diffractometer (XRD, Bruker D8 Advance, Bruker AXS, Saarbruchen, Germany). The specific surface area and pore characterization were measured using N_2_ adsorption and desorption data at −196 °C using Micromeritics ASAP 2020 M (Micromeritics, Norcross, GA, USA). Raman spectra were obtained using a laser micro-Raman spectrometer (Renishaw, Gloucestershire, UK) to characterize the lattice structure of the carbon materials.

### 2.4. Preparation of ABPC Electrode Sheet

Firstly, the electrode materials need to be prepared. In the three-electrode system, 8 mg porous carbon material, 1 mg carbon black, and 1 mg polytetrafluororthylene binder were mixed in a mortar (i.e., maintaining a ratio of 8:1:1), adding an appropriate amount of anhydrous ethanol, then fully mixed, and ground for 10 min until the mixture was flake solid. The thoroughly ground mixture was coated on a 1 × 2 cm^2^ nickel foam sheet. The obtained sample was compressed with a press at 15 MPa for 2 min and collected to obtain the target working electrode. The mass load of the active material on each nickel foam electrode is about 5 mg cm^−2^.

The electrochemical measurements were performed using an electrochemical workstation (CHI760E, Shanghai, China). In the three-electrode system, 6 M KOH solution was used as the electrolyte, and Pt electrode and HgO/Hg electrodes were used as the counter and reference electrodes, respectively. In the two-electrode system, two identical electrode materials were used as positive and negative electrodes, and a polypropylene diaphragm was selected for symmetric capacitor assembly.

### 2.5. Electrochemical Measurements

The electrochemical measurements included cyclic voltammetry (CV), constant current charge/discharge (GCD), electrochemical impedance spectroscopy (EIS), and cycling performance tests [33].

In the three-electrode system, the specific capacitance *C*_1_ (F g^−1^) of GCD can be calculated with Equation (1):

(1) $$C_{1}=I\times ∆t/m\times ∆V$$

where *I* (A) is the discharge current, ∆*t* (s) is the discharge time, *m* (g) is the mass of the electrode material, and ∆*V* is the potential window.

In the two-electrode system, the specific capacitance of the assembled symmetrical capacitor *C*_2_ (F g^−1^) can be calculated with Equation (2):

(2) $$C_{2}=2\times I\times ∆t/m\times ∆V$$

where *I* (A) is the discharge current, ∆*t* (s) is the discharge time, *m* (g) is the mass of the electrode material, and ∆*V* is the potential window.

The energy density *E* (Wh kg^−1^) and power density *P* (W kg^−1^) can be calculated with Equations (3) and (4):

(3) $$E=\frac{1}{2\times 3.6}\times C_{1}\times ∆V^{2}$$

(4) $$P=\frac{E}{∆t}\times 3600$$

where ∆*V* (V) is the potential and ∆*t* (s) is the discharge time.

## 3. Results and Discussion

### 3.1. Morphology and Composition of the Prepared Porous Carbon

**Figure 1:**
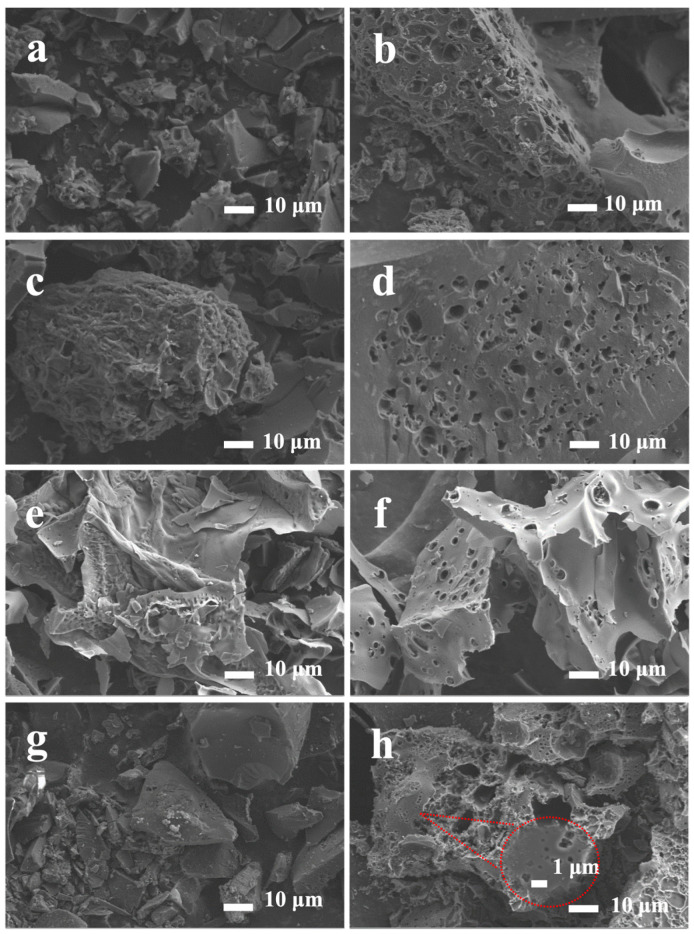
SEM images of (**a**) BC, (**b**) ABC, (**c**) BPC-1, (**d**) ABPC-1, (**e**) BPC-2, (**f**) ABPC-2, (**g**) BPC-4, (**h**) ABPC-4.

As shown in Figure 1a, the pre-carbonized product BC without potassium hydroxide (KOH) activation had a complete hollow-tube structure and essentially no porous structure on its surface. In comparison, the porous structure on the surface of ABC was visible, as is the spongy microstructure. As shown in Figure 1b, its porous carbon was widely distributed, with pore sizes ranging from tens to hundreds of nanometers. The formation of these porous structures emerged as a result of the separation of hydrophobic carbon from the original structure during the potassium hydroxide (KOH) activation process [34]. Similar macropores also appeared in Figure 1d,f,h; the activated porous structure was well preserved when the biomass was doped with nitrogen, proving that the biomass doping used in this experiment had no effect on the activated porous structure. Thermogravimetric analysis (TGA) was used to investigate the changes during the pyrolysis of all mixtures, including broccoli and pig skin alone. When the temperature was greater than 500 °C, all mixtures basically no longer underwent thermochemical changes and mass loss, which indicated that all mixtures were completely carbonized to form carbon materials (Figure S1).

**Figure 2:**
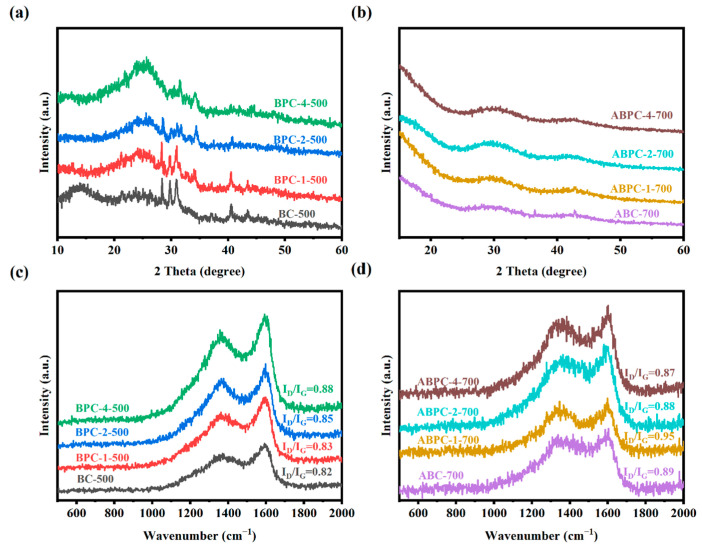
XRD spectra of (**a**) pre-carbonization products, (**b**) high temperature activated porous carbon. Raman patterns of (**c**) pre-carbonization products, (**d**) high temperature activated porous carbon.

Figure 2a,b depicted X-ray diffraction images from various research groups. Figure 2a shows the pre-carbonized products under varied mixed biomass conditions, namely, the diffraction pattern without being activated by potassium hydroxide. Figure 2b shows the product’s diffraction pattern after the sample has been activated. It was noticed that the four types of pre-carbonized biochars had modest diffraction peaks at 2θ angles of 30° and 40°, which primarily correspond to crystal planes (011), (101), and (200). The crystalline properties of carbon materials in pre-carbonized samples were highly limited and did not reveal visible crystal structure based on the crystal plane information and the smoothness of the entire XRD pattern [35]. Following additional elution and high-temperature carbonization, corresponding to the diffraction patterns of four types of samples in Figure 2b, it was discovered that the peak value at low angle increases significantly, and the curve as a whole is complete and smooth. Two basic causes underlay this. First, after activation, a large number of micropore structures were developed [36], which correlated to an increase in low-angle diffraction peaks. Second, carbon dominates the entire structure, resulting in a smooth amorphous diffraction peak pattern. Connecting the elemental analysis of the experimental samples with the XRD images, as shown in Table 1, explained the difference in diffraction peaks before and after activation, which was primarily due to the loss of some doping elements during high-temperature carbonization, particularly the difference between ABPC and BPC: the complete loss of sulfur and the reduction of nitrogen after activation, which made the diffraction results more likely to show the amortization.

The Raman spectra of the produced material are shown in Figure 2c,d, which consists mostly of two characteristic peaks, the D band near 1350 cm^−1^ and the G band near 1590 cm^−1^. The defect level and degree of biochar disorder can be determined by fitting the ratio of D peak to G peak area (I_D_/I_G_) on the Raman spectra [37]. The higher the ratio, the more defective the biochar materials. The I_D_/I_G_ values of various materials could be calculated. Compared with Figure 2c,d, it was discovered that the I_D_/I_G_ of activated carbon activated by potassium hydroxide is relatively high, indicating that the defect degree of the material had increased, and the meaning of the defect was explained. The C atom, in general, referred to covalent coupling or marginalization, which could also indicate that the porous structure of activated carbon increased the defect and disorder degree of the entire activated carbon. While the I_D_/I_G_ value of Figure 2c was also high, this could have been due to more sp^2^ hybrid C atoms being coupled with nitrogen during nitrogen doping [38].

**Figure 3:**
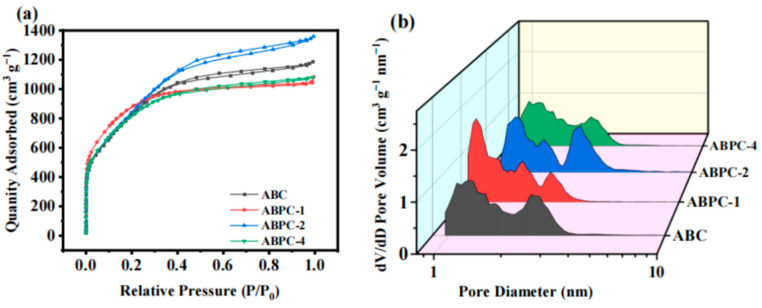
(**a**) N_2_ adsorption and desorption isotherms, (**b**) Pore size distribution diagram.

The N_2_ adsorption-desorption isotherm and pore size distribution were used to evaluate the structural properties of porous carbon produced from multi-source nitrogen-rich food waste (Figure 3). From the linear shape of the adsorption isotherm, the adsorption capacity of biochar material increased rapidly at lower relative pressure and then tended to saturate. This was a typical microporous filling phenomenon on microporous adsorbents, and the saturated adsorption value is approximately equal to the filling volume of the micropores. Furthermore, after pyrolysis activation, all adsorption and desorption curves (Figure 3a) of nitrogen-rich food waste with different components had a cleaning hysteresis loop in the range of P/P_0_ of 0.4–0.9, which are all expressed as I and IV combined isotherms [39]. This adsorption curve also supported the porous structure of the activated material. Table 2 shows the specific surface area and pore volume of each material. Because all of the samples had extremely high specific surface area (>3000 m^2^ g^−1^), the effect of collagen protein addition on the specific surface area was insignificant. When pigskin was added to the biomass raw material, the micropore volume of porous carbon was reduced, and SEM observations were consistent. This was because, under KOH activation conditions, collagen is transformed into NH_3_, resulting in greater pore size (Figure 3b) and more micropores and structures [23]. The pore size distribution plot showed that it was mostly dispersed between 1 and 4 nm. Because an increase in mesopores and macropores helped to increase ion transport channels and decrease electrolyte transport resistance, porous carbon derived from ABPC-4 nitrogen-rich food waste with micropores, mesopores, and macropores was beneficial to ion or reactant diffusion.

**Figure 4:**
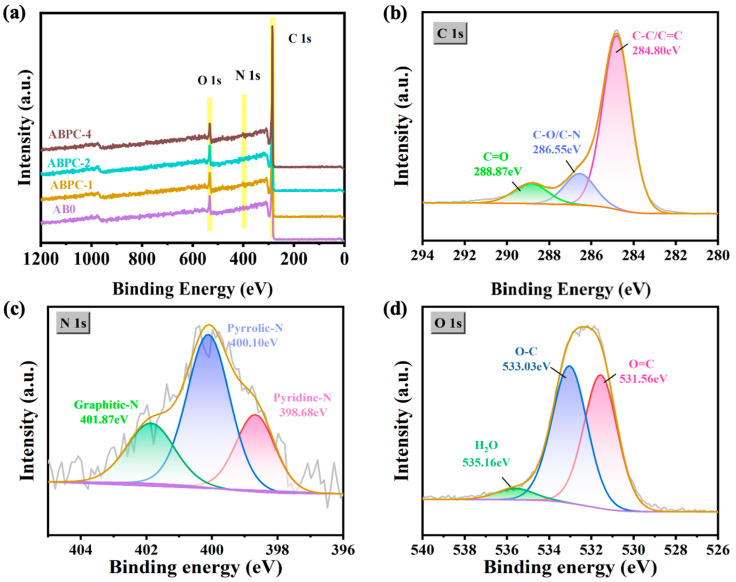
(**a**) XPS measurement spectra of samples ABC, ABPC-1, ABPC-2, and ABPC-4, (**b**–**d**) Fine spectra of C 1s, N 1s, and O 1s of sample ABPC-4.

The surface element content and structure of carbon compounds produced from endogenous nitrogen-rich biomass were evaluated using XPS analysis (Figure 4). Figure 4a mainly analyzed the bond energy distribution of biochar after activation. From the XPS images of the three characteristic peaks, it could be seen that the main doping elements of the carbon-based material were oxygen and nitrogen. Figure 4b–d shows the C 1s and O 1s XPS spectra of ABPC-4, including three C 1s characteristic peaks at approximately 284.8, 286.55, 288.87 eV and three O 1s characteristic peaks at 531.56, 533.03, and 535.16 eV, representing C-C/C=C, C-O/C-N, C=O and O=C, O-C, and H_2_O bonds, respectively [40]. It is worth mentioning that the combination of carbon, nitrogen, and carbon results in the formation of the nitrogen-doped carbon structure via amino acid breakdown [23]. The N-doped carbon structure was also confirmed by the N 1s XPS photoelectron spectra of ABPC-4, which contained pyridine-N, pyrrolic-N, and graphitic-N at 398.68, 400.10, and 401.87 eV, respectively (Figure 4c) [41].

### 3.2. Electrochemical Performance of Three Electrodes

**Figure 5:**
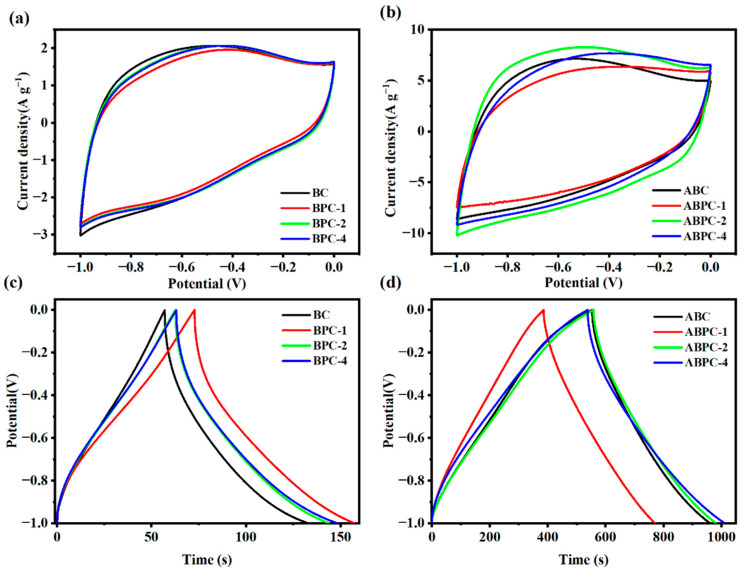
(**a**,**b**) CV curves of pre-carbonization biochar and porous carbon at a scanning rate of 20 mV s^−1^, (**c**,**d**) GCD curve of pre-carbonization biochar and porous carbon at current density 1 A g^−1^.

The electrochemical performance of biochar as electrode material was evaluated in a three-electrode system with 6 M KOH as the electrolyte. As shown in Figure 5a,b, the CV plots of pre-carbonization products and high-temperature activated porous carbon with different combinations of N-enriched biomass at a scan rate of 20 mV s^−1^ were quasi-rectangular. Compared with the pre-carbonization product of collagen-rich biomass (Figure 5a), the CV curves of the porous carbon after high-temperature activation were closer to the rectangular shape with the characteristics of a double-layer capacitor (Figure 5b). The charging and discharging times of the materials before and after activation were also investigated, and the discharging time increased from 60 s to 400 s after KOH activation (Figure 5c,d). This was because the chemical activation gave the material an ultra-high specific surface area and rich pore structure, which accelerated the transport and diffusion of ions [42]. The GCD curve showed a quasi-triangular shape, which was not strictly symmetric due to the pseudo-capacitance provided by the N and O atoms. The specific capacitance of ABPC-4 was calculated to be the largest at a current density of 1 A g^−1^, which was 473.03 F g^−1^. This was because the additional N, O atoms doping could change the charge mobility, which improved the surface wettability and specific capacitance of the electrode material [43].

**Figure 6:**
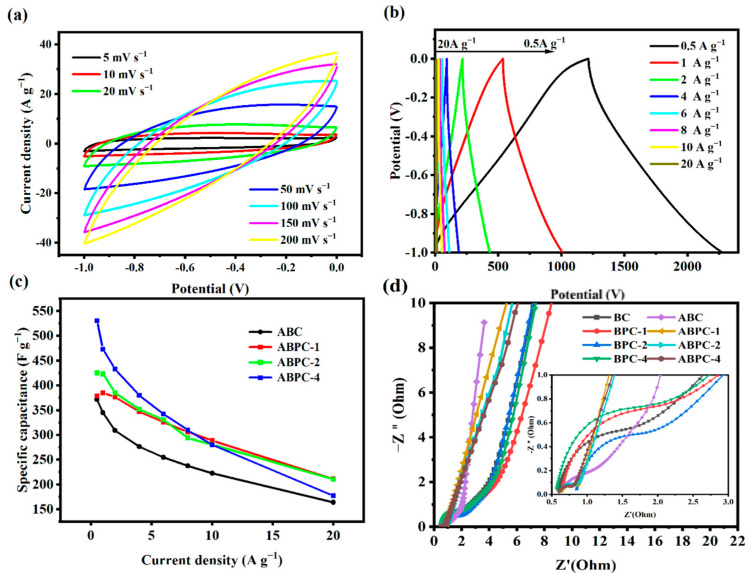
(**a**) CV curves of samples ABPC-4 at different scanning rates, (**b**) GCD curves of sample ABPC-4 at different current densities, (**c**) Specific capacitance of nitrogen-rich biomass-derived porous carbon, (**d**) Impedance profile of nitrogen-rich biomass-derived porous carbon.

Furthermore, cyclic voltammetric curves at various scanning rates were tested to reveal the capacitance performance of ABPC-4 (Figure 6a). ABPC-4 exhibited nearly perfect double-layer capacitance behavior at a low scanning rate of 5–20 mV s^−1^. The pseudocapacitors produced by pyridine-N and pyrrole-N result in a broad redox peak in the range of −0.4 to −0.7 V. The CV curve became deformed as the scanning rate increased, which could be owing to the restricted structure of quaternary-N and pyridine-N oxides, which inhibited electron transmission in N-doped porous carbon [44]. Figure 6b depicted additional charge and discharge experiments on ABPC-4 at various current densities. When the current density was increased to 20 A g^−1^, the GCD curve remained almost triangular, indicating that the ABPC-4 had good rate performance [45]. The GCD curve was used to compute the specific capacitance of porous carbon generated from nitrogen-rich biomass, as illustrated in Figure 6c. When the current density was 1 A g^−1^, the specific capacitance of the ABPC-4 was 473.03 F g^−1^. Specific capacitances of ABPC-1, ABPC-2, ABPC-4, and ABC fell to 55.73%, 55.67%, 49.6%, and 33.40% of the original capacitance, respectively, as the current density increased from 0.5 A g^−1^ to 20 A g^−1^ (Figure 6c).

Figure 6d depicts the impedance spectra of pre-carbonized products made from various combinations of nitrogen-rich biomass and high-temperature-activated porous carbon. The slope of pre-carbonization products of collagen-rich biomass was too steep in the low-frequency zone, indicating that its corresponding series resistance was too strong. However, after high-temperature activation, the porous carbon was almost perpendicular to Z′, indicating that double-layer charge storage was dominant [42]. Although ABC and other samples (such as ABPC-2 and ABPC-4) had similar specific surface areas and pore size distributions, they differ in material composition and surface chemical properties. Specifically, ABC samples without the addition of pig skin had different nitrogen content. This chemical property affected the conductivity and electrochemical reactivity of the material. The uniform pore structure and high nitrogen and oxygen content of the samples ABPC added with pig skin can improve the wettability of the electrode material, further improve the effective active area of the electrode material, and minimize its R_ct_. This resulted in different behaviors in EIS [46]. ABPC-4 had the smallest semicircle diameter in all high-frequency ranges before and after activation, demonstrating that ABPC-4 had lower charge transfer resistance, which was conducive to the rapid transfer and diffusion of electrons.

### 3.3. Electrochemical Performance of Two Electrodes

**Figure 7:**
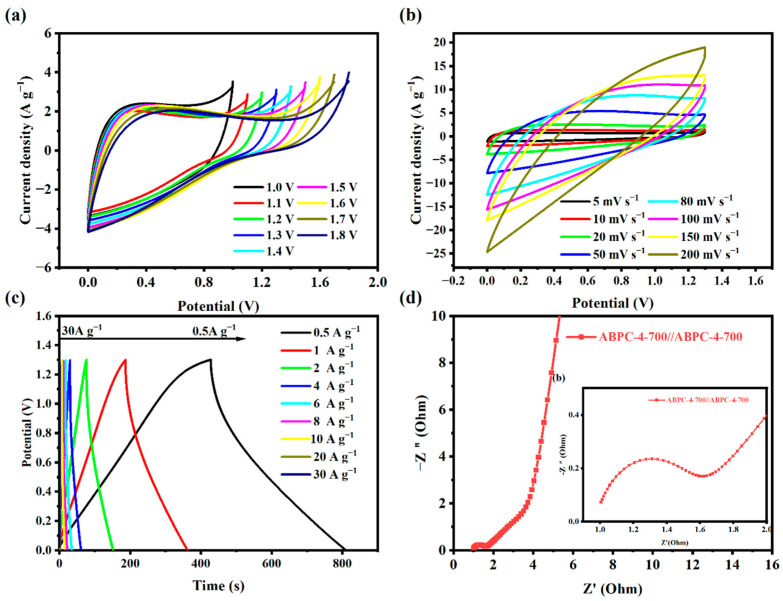
(**a**) CV curves of ABPC-4//ABPC-4 at different voltage windows of 1–1.8 V, (**b**) CV curves of ABPC-4//ABPC-4 at different scanning rates of 5–200 mV s^−1^, (**c**) GCD curves at different densities from 0.5 to 30 A g^−1^, (**d**) Impedance spectra (Nyquist plots).

To better assess the practical application capability of ABPC-4, an ABPC-4//ABPC-4 symmetrical supercapacitor was created using a 6 M KOH electrolyte. During the charging process, K^+^ and OH^−^ ions in the electrolyte migrated to the cathode and anode, respectively, forming a double layer at the electrode-electrolyte interface [47]. Once charging was complete, the positive and negative charges on the electrodes were attracted to the oppositely charged ions in the solution, stabilizing the double layer and establishing a potential difference between the electrodes, which represented the stored energy in the supercapacitor [48]. During discharge, charge migration on the electrodes generated current, while ions in the solution returned to the electrolyte [49]. The CV curves of varied voltage windows of two electrodes at a scanning rate of 20 mV s^−1^ are shown in Figure 7a. At high voltages of each CV, the current began to increase exponentially, which was usually caused by the oxygen evolution reaction, which resulted from the oxidation of water on the electrode surface. When the potential exceeded a certain threshold (the oxygen evolution potential), water began to decompose on the electrode surface, producing oxygen. This phenomenon led to a sharp rise in current, appearing as the exponential growth segment in the CV curve [50]. The exponential disappearance in subsequent scans may be caused by factors such as surface passivation, electrode material modification, or changes in the electrolyte environment [51,52]. The CV curves maintained consistent symmetry when the voltage window grew from 0 to 1.0 V to 0 to 1.3 V. Figure 7b showed CV curves at various scanning rates. The two electrodes of ABPC-4//ABPC-4 displayed a rectangular curve with acceptable symmetry at a scanning rate of 5–50 mV s^−1^. When the scanning rate was increased to 100–200 mV s^−1^, the rectangle of the curve became distorted due to the pseudo charge transfer resistance and ion delay present in the micropores [53]. Figure 7c shows the charge-discharge curve’s features. The specific capacitance of ABPC-4//ABPC-4 was 273.41 F g^−1^ at 1 A g^−1^ current density. Furthermore, the GCD curve had obvious symmetry under different current densities, and almost no distortion was observed, indicating that it had good charge and discharge reversibility and rate ability. The completed symmetrical supercapacitor’s Nyquist diagram indicated that the low-frequency zone was roughly vertical, and it subsequently transitioned from the 45° Warburg intermediate frequency region to a semicircle [54]. The R_s_ of ABPC-4 as a two-electrode device was 0.59 Ω, suggesting that it had a low load transfer resistance (Figure 7d).

### 3.4. Cycle Efficiency of Supercapacitors

**Figure 8:**
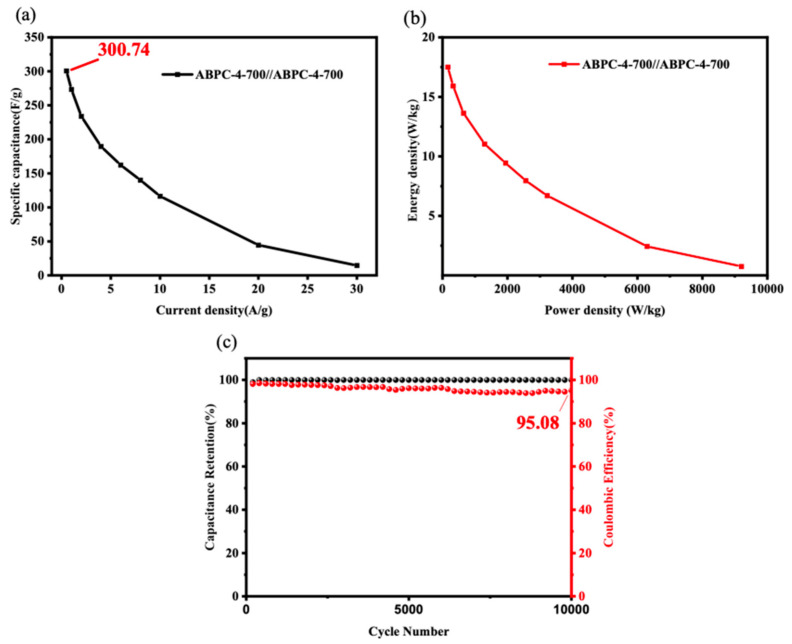
(**a**) The Rogone diagram of ABPC-4; (**b**) Magnification performance graph of ABPC-4; (**c**) Cycling stability of sample ABPC-4 in a three-electrode system.

The Ragone diagram of the best charging and discharging material ABPC-4 was shown in Figure 8a, which was used to calculate the trade-off connection between specific energy and specific power of electrode materials. The energy-power relationship of the mixed biomass activated carbon material in this investigation was superior to that of the standard battery in the two-electrode experiment. In general, when the specific power of traditional batteries, such as lithium batteries, reached a critical value, its specific energy rapidly decreased, and it was unable to achieve a stable condition under diverse energy requirements in actual applications [55]. However, under experimental conditions, the supercapacitor made of biomass-activated carbon material exhibited a gentle inverse correlation between specific energy and specific power, implying greater energy stability and application potential. Furthermore, based on the numerical analysis, the specific power of the ABPC-4 biochar material could be close to 10,000 W kg^−1^ at a current density of 30 A g^−1^, demonstrating the material’s excellent performance.

GCD charge-discharge measurements under 10,000 cycles were used to determine the cyclic stability of ABPC-4, as illustrated in Figure 8c. The capacitance material composed of ABPC-4 not only retained 100% capacitance after a long-term GCD cycling, indicating that the charging and discharging processes had no effect on the material’s capacitance characteristics, but also had a coulombic efficiency of more than 95% after 10,000 cycles. The rate performance was also an index that reflected the electrode’s charge and discharge ability under varied loads, as illustrated in Figure 8b. Began with a modest current density of 0.5 A g^−1^ and gradually increased the test magnification until it stopped at 30 A g^−1^, obtaining the specific capacitance change curve. The specific capacitance at 0.5 A g^−1^ reached 300.74 F g^−1^ in this two-electrode setup, and the decreasing trend steadily slowed as the rate increased. On the whole, the two electrodes of the ABPC-4 could have high capacity, good cycle stability, and rate performance. The performances of some recent biomass electrode materials reported in the literature are listed in Table 3, among which ABPC-4 exhibited great advantages in both SSA and electrochemical properties.

## 4. Conclusions

In summary, the nitrogen-doped porous carbon was successfully produced by pre-carbonization and activation using mixed broccoli and nitrogen-rich pigskin. The effect of different mixing ratios of raw materials on the microstructure, elemental content, and electrochemical properties of the nanoporous biochar was investigated. The ABPC-4 electrode possesses a super high specific surface area of 3030.2 m^2^ g^−1^, as well as plentiful nitrogen (2.19 wt%) and oxygen content (11.75 wt%). In a three-electrode system, the ABPC-4 showed an outstanding specific capacitance of 473.03 F g^−1^ at a current density of 1.0 A g^−1^, outperforming most electrode materials prepared solely from biomass. Meanwhile, at the current density of 30 A g^−1^, the specific capacitance of ABPC-4//ABPC-4 was 273.41 F g^−1^ in the symmetric supercapacitor, and it exhibited excellent cycle stability at the current density of 10 A g^−1^ (the capacitance retention rate was 95.08% after 10,000 cycles). Furthermore, the power density was 161.85 W kg^−1^ when the maximum energy density was 17.51 Wh kg^−1^, demonstrating that ABPC-4, as an electrode material, had excellent energy storage performance. This study not only comes up with a low-cost and ecologically benign source to prepare supercapacitor electrodes but also helps us have a better understanding of the effect of element doping on energy storage behavior.

## Figures and Tables

**Table 1:** Elemental analysis results of biomass materials.

Samples	C [%]	H [%]	N [%]	O [%]	S [%]	H/C Ratio	O/C Ratio	(O + N)/C
Broccoli	41.67	7.094	5.57	44.83	0.836	0.1702	1.0758	1.2095
Pigskin	49.42	8.01	14.64	27.93	0	0.1621	0.5652	0.8614
BC	61.18	2.919	6.95	28.375	0.576	0.0477	0.4638	0.5774
BPC-1	61.03	3.068	8.01	27.469	0.423	0.0503	0.4501	0.5813
BPC-2	60.32	3.092	8.72	27.682	0.186	0.0513	0.4589	0.6035
BPC-4	62.98	3.464	9.67	23.886	0	0.0550	0.3793	0.5328
ABC	85.62	1.743	0.86	11.777	0	0.0204	0.1375	0.1476
ABPC-1	80.77	2.381	3.59	13.259	0	0.0295	0.1642	0.2086
ABPC-2	87.72	1.339	0.86	10.081	0	0.0153	0.1149	0.1247
ABPC-4	84.26	1.803	2.19	11.747	0	0.0214	0.1394	0.1654

**Table 2:** Specific surface area and pore volume of samples.

Samples	S_BET_ ^a^ (m^2^ g^−1^)	S_micro_ ^b^ (m^2^ g^−1^)	V_pore_ ^c^ (cm^3^ g^−1^)	V_micro_ ^d^ (cm^3^ g^−1^)	Pore Size ^e^ (d, nm)
BPC-1	20.4	—	0.0042	0.001	—
BPC-2	21.0	—	0.0040	—	—
BPC-4	20.2	—	0.0037	—	—
BC	1.1	—	0.0036	—	—
ABPC-1	3065.3	908.9	1.6752	0.3755	2.2575
ABPC-2	3147.0	1065.8	2.1035	0.4839	2.3102
ABPC-4	3030.2	377.7	1.6491	0.1275	2.2969
ABC	3088.6	1836.5	1.8326	0.6857	2.252

^a^ BET surface area; ^b^ Micropore (<2 nm) surface area calculated; ^c^ Total pore volume obtained at P/P_0_ = 0.99; ^d^ Micropore volume calculated by t-plot method; ^e^ Mesopore volume calculated by BJH method.

**Table 3:** Comparison of SSA and electrochemical properties between ABPC-4 and other biomass electrode materials.

Biomass Electrode Materials	SSA (m^2^ g^−1^)	Specific Capacitance (F g^−1^)	Current Density (A g^−1^)	Specific Capacitance of Two-Electrode System (F g^−1^)	Current Density of Two-Electrode System (A g^−1^)	Energy Density (Wh kg^−1^)	Power Density (W kg^−1^)	Cycle Stability (%)	References
ABPC-4	3030.2	473.03	1	273.41	1	17.51	161.85	95.08/10,000 cycles	This work
Rapeseed meal (N-P co-doped)	3765.7	460	1	245.0	1	10.1	625	83.9/10,000 cycles	[56]
Gelatin (ZnCl_2_ hybrid)	1336	337.6	0.5	286.2	0.5	120.1	450	95.6/10,000 cycles	[57]
Soybean straw	2266.19	380.5	0.5	218	1	13.2	52.03	73.9/10,000 cycles	[58]
Hyphaene fruit shell (multi-heteroatom doped)	197.99	337	0.7	159.78	1	28.5	330	82/3000 cycles	[59]

## Data Availability

Data are contained within the article and Supplementary Materials.

## Supplementary Materials

The following supporting information can be downloaded at: https://www.mdpi.com/article/10.3390/nano14161368/s1, Figure S1: (a) TG and (b) DTG curves of broccoli, pigskin, and all mixture. Figure S2: EIS simulation equivalent circuit. Figure S3: Schematic diagram of preparation process of porous carbon from food. Figure S4: Two-electrode device in both ON and OFF conditions with timing.
