# Supplementary material for: In Situ, Nitrogen-Doped Porous Carbon Derived from Mixed Biomass as Ultra-High-Performance Supercapacitor
Source: Nanomaterials (Basel). 2024 Aug 21;14(16):1368. doi: 10.3390/nano14161368 (PMC11357087; doi:10.3390/nano14161368)
Supplement: Supplementary file 1 [file nanomaterials-14-01368-s001.zip › nanomaterials-3151670-supplementary.pdf]

Supporting Information

In Situ, Nitrogen-Doped Porous Carbon Derived from Mixed Biomass as Ultra-High-Performance Supercapacitor

Yuqiao Bai [1], Qizhao Wang [1], Jieni Wang [1,2], Shuqin Zhang [1,2], Chenlin Wei [1,2], Leichang Cao [1,2,*] and Shicheng Zhang [3]

1 Miami College, Henan University, Kaifeng 475004, China

2 College of Chemistry and Molecular Sciences, Henan University, Kaifeng 475004, China

3 Shanghai Key Laboratory of Atmospheric Particle Pollution and Prevention (LAP3), Department of Environmental Science and Engineering, Fudan University, Shanghai 200433, China

* Correspondence: clch666@henu.edu.cn

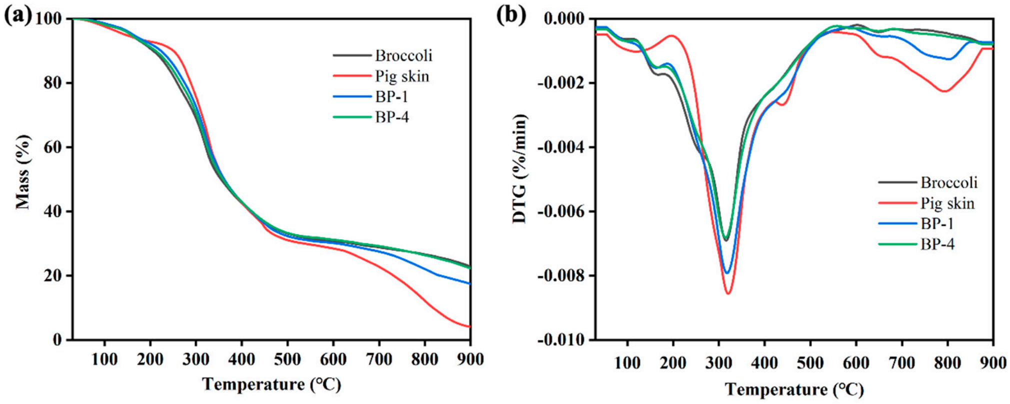

Figure S1. (a) TG and (b) DTG curves of broccoli, pigskin, and all mixture.

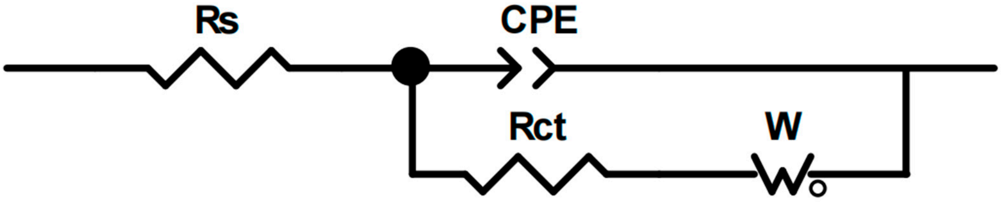

Figure S2. EIS simulation equivalent circuit.

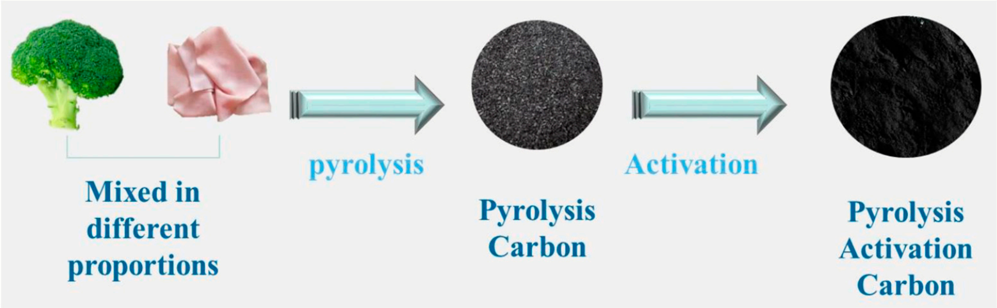

Figure S3. Schematic diagram of preparation process of porous carbon from food.

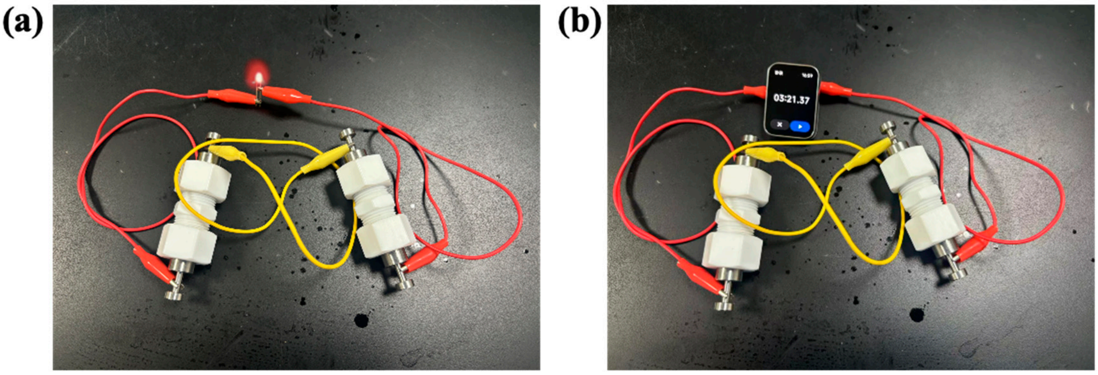

Figure S4. Two-electrode device in both ON and OFF conditions with timing.
